# Fatal aortoesophageal fistula arising from a remodeled descending aorta after repair of acute type A intramural hematoma

**DOI:** 10.1016/j.xjse.2026.100125

**Published:** 2026-04-08

**Authors:** Satoshi Asada, Shinsuke Kotani, Ryumon Matsumoto, Ryusuke Hamada, Yusuke Imamura, Naoya Miyashita, Genichi Sakaguchi

**Affiliations:** Department of Cardiovascular Surgery, Kindai University Hospital, Osaka, Japan

**Figure:**
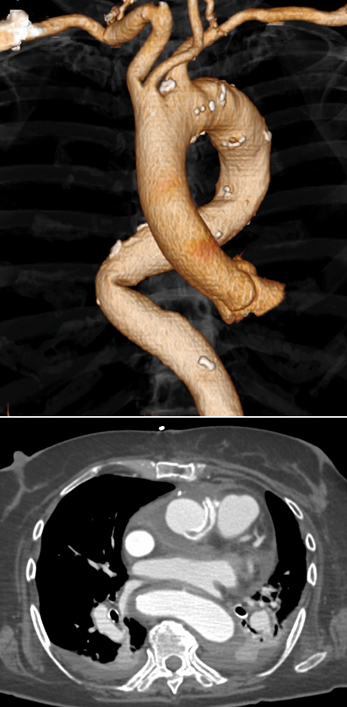
CT demonstrated a tortuous remodeled descending aorta with AEF after type A IMH surgery.

**Central Message**

AEF may occur even in a remodeled descending aorta after repair of acute type A IMH, particularly in patients with severe aortic tortuosity.

Aortoesophageal fistula (AEF) is a rare but catastrophic condition associated with extremely high mortality. It is most commonly reported as a secondary complication related to thoracic aortic aneurysmal dilatation, prosthetic graft implantation with felt reinforcement, or prior thoracic endovascular aortic repair (TEVAR).^1^^,^^2^ Despite advances in surgical and endovascular techniques, the prognosis of AEF remains poor.^1^^,^^2^

Acute aortic syndromes include classic aortic dissection and intramural hematoma (IMH), the latter characterized by hemorrhage within the aortic media without an identifiable intimal tear or patent false lumen in the ascending aorta.^3^ Acute type A IMH is generally managed surgically according to current European Association for Cardio-Thoracic Surgery/Society of Thoracic Surgeons guidelines.^3^ Although the primary goal of surgical treatment for type A aortic dissection is elimination of proximal pathology and promotion of favorable remodeling of the distal aorta,^4^ the long-term clinical implications of remodeled distal aortic segments remain incompletely understood.

We report an extremely rare case of fatal AEF arising from a remodeled descending thoracic aorta after hemiarch replacement for acute type A IMH in a patient with marked aortic tortuosity.

## Case

An 80-year-old woman had previously undergone Y-graft replacement for an abdominal aortic aneurysm at another institution and had been followed for a proximally thrombosed anastomotic pseudoaneurysm. She presented with chest pain and was emergently transferred to our hospital, where contrast-enhanced computed tomography (CT) established the diagnosis of acute type A intramural hematoma (IMH).

**Figure 1:**
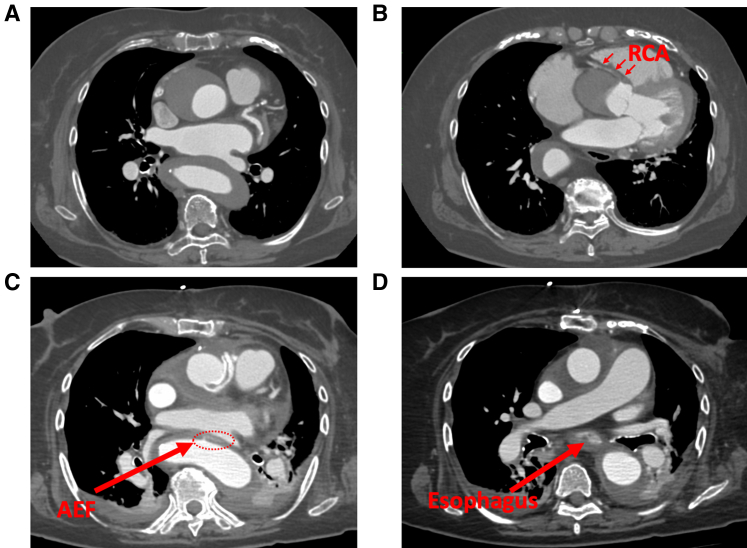
Preoperative and postoperative contrast-enhanced CT images. A and B, Preoperative CT images obtained at the onset of acute type A IMH. C and D, Postoperative CT images obtained at the onset of AEF. A, Circumferential thickening of the ascending aortic wall consistent with type A IMH. The ascending aortic diameter measured 45 mm with hematoma thickness of 20 mm. The descending aortic diameter at the future fistula site was 27 mm with hematoma thickness of 5 mm. B, Long-segment narrowing of the RCA near its origin, suggesting coronary involvement. C, Focal contrast extravasation from the descending thoracic aorta into the esophagus, consistent with AEF. At this level, the aortic diameter measured 25 mm with hematoma thickness of 2 mm, indicating continued remodeling without aneurysmal dilatation. The dashed circle indicates the esophagus at the compressed segment. D, Contrast pooling within the esophageal lumen proximal to the fistula, further supporting the diagnosis of AEF. *AEF*, Aortoesophageal fistula; *RCA*, right coronary artery.

**Figure 2:**
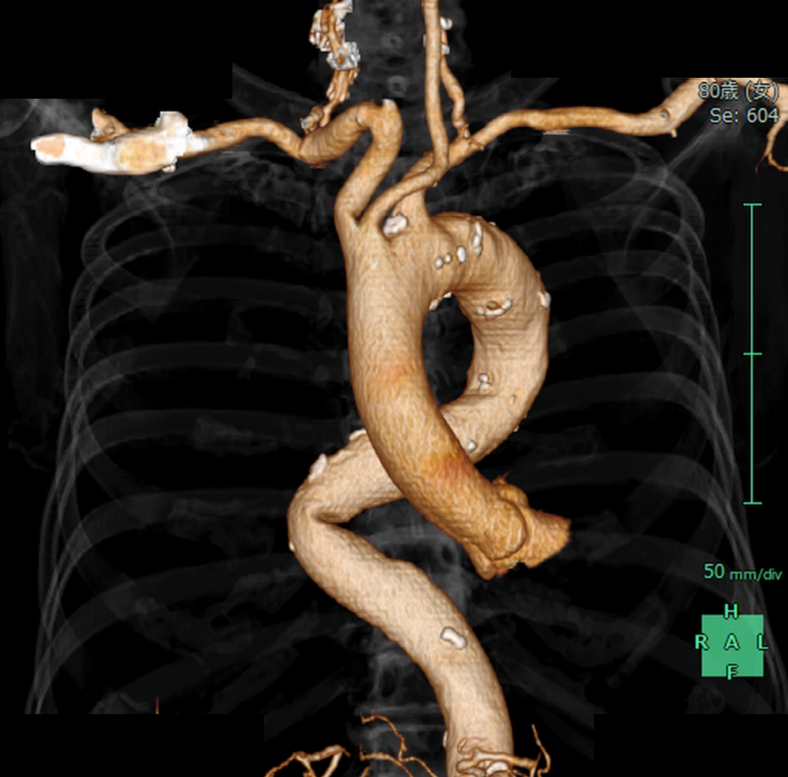
Three-dimensional reconstructed CT demonstrating marked tortuosity of the descending thoracic aorta. The descending thoracic aorta courses behind the left atrium and is in close proximity to the esophagus, suggesting a potential site of chronic mechanical compression.

Contrast-enhanced CT revealed a thrombosed false lumen in the entire dissected aorta without an identifiable entry tear. The IMH was markedly thickened, indicating extensive involvement of the aortic wall. The ascending aortic diameter measured 45 mm with hematoma thickness of 20 mm (Figure 1, *A*). The IMH extended from the level of the right coronary artery (RCA) origin to the superior mesenteric artery, without penetrating aortic ulcer or ulcer-like projection. The descending thoracic aorta showed marked tortuosity, coursing behind the left atrium (Figure 2). Preoperative CT also demonstrated long-segment narrowing of the RCA near its origin (Figure 1, *B*), although complete flow interruption was not observed, and coronary malperfusion was not strongly suspected preoperatively. Severe aortic regurgitation was also present.

Emergency hemiarch replacement was performed. During selective antegrade cardioplegia, intimal disruption involving the RCA origin was identified intraoperatively, and the RCA root was ligated with saphenous vein graft bypass to the RCA segment number 1. No entry tear was identified in the ascending or arch segments, and the pathology was considered consistent with retrograde DeBakey type IIIb morphology. The aortic valve was preserved with commissural suspension, and postoperative echocardiography demonstrated mild residual aortic regurgitation with preserved left ventricular function (with an ejection fraction of 50%). The postoperative course was initially stable with satisfactory blood pressure control. Follow-up CT demonstrated that the residual IMH remained thrombosed with progressive favorable remodeling of the descending thoracic aorta. Because of delayed recovery of swallowing function, the patient required enteral nutrition via a standard nasogastric tube from the day of surgery, which was replaced with a soft enteral feeding tube on postoperative day 19.

**Figure E1:**
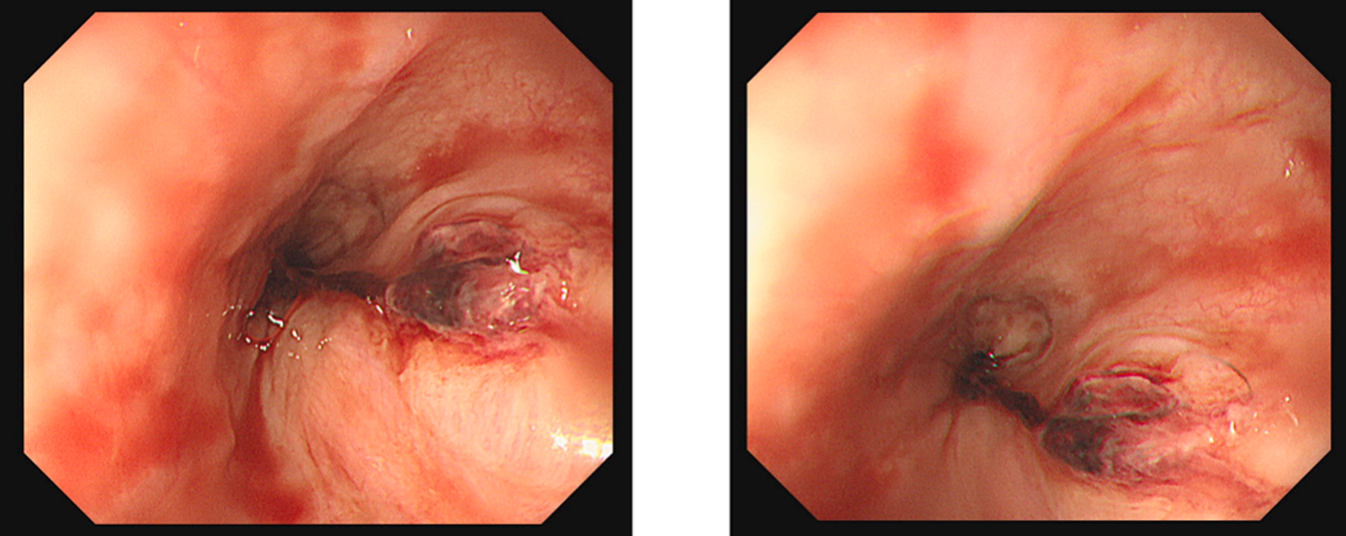
Endoscopic findings. Upper gastrointestinal endoscopy demonstrating a submucosal protrusion with ulceration in the mid-to-distal esophagus, corresponding to the site of the AEF. Of note, no linear ulceration or mucosal injury suggestive of enteral feeding tube–related mechanical stress was identified anywhere in the esophagus.

Approximately 1 month after surgery, the patient suddenly developed massive hematemesis without preceding chest pain or sentinel bleeding. Emergency upper gastrointestinal endoscopy demonstrated a submucosal protrusion with ulceration in the mid-to-distal esophagus (Figure E1), without linear ulceration suggestive of tube-related mucosal injury. Contrast-enhanced CT demonstrated focal contrast extravasation from the descending thoracic aorta into the esophagus, establishing the diagnosis of AEF (Figure 1, *C*). The descending aortic diameter at the affected segment measured 27 mm with hematoma thickness of 5 mm before onset and 25 mm with thickness of 2 mm at the time of AEF, indicating continued remodeling without aneurysmal dilatation (Figure 1, *A* and *C*). CT also demonstrated contrast pooling within the esophageal lumen proximal to the fistula (Figure 1, *D*). Although salvage TEVAR was considered as a potential lifesaving intervention, catastrophic rebleeding occurred immediately after CT imaging, leading to cardiopulmonary arrest before endovascular treatment could be initiated. Despite resuscitative efforts, the patient could not be salvaged.

## Discussion

AEF remains one of the most devastating complications of thoracic aortic disease. Without treatment, mortality approaches 100%, and even with aggressive surgical or endovascular interventions, reported in-hospital mortality ranges from 30% to 60%, with 1-year survival rates below 30%.^1^^,^^2^ Most reported cases of secondary AEF are associated with thoracic aneurysmal dilatation, prosthetic graft material, felt reinforcement, or prior TEVAR.^1^^,^^2^ In contrast, AEF unrelated to prosthetic material or aneurysmal expansion is extremely rare.^5^^,^^E1^

In the present case, fistula formation was likely multifactorial. Marked tortuosity of the descending thoracic aorta created focal compression of the esophagus between the aorta and the posterior wall of the left atrium. Age-related elongation and loss of aortic elasticity may predispose to such tortuosity and contribute to acute aortic syndromes.^E2^ Of note, prior CT imaging obtained 1 year before the onset of IMH during oncologic surveillance had already demonstrated the same marked tortuosity of the descending thoracic aorta, suggesting that this was a preexisting anatomic feature rather than a newly developed acute change.

Prolonged nasogastric instrumentation may have increased mucosal vulnerability. However, the absence of linear ulceration on endoscopy suggests that tube-related injury alone was unlikely to be the primary mechanism. The endoscopic and CT findings instead support a focal process driven by anatomic compression and local vulnerability of the aortic wall.

In retrograde type IIIb dissection or IMH, the optimal surgical strategy remains controversial. Although hemiarch replacement is often selected to minimize operative risk, more aggressive approaches such as total arch replacement with or without a frozen elephant trunk have been advocated to promote distal remodeling and reduce late reintervention.^4^ Acute type A IMH is also a distinct clinicopathologic entity from classic dissection and has been reported to have distinct operative characteristics and outcomes.^E3^ In the present case, the primary entry was not clearly identified, and severe aortic regurgitation precluded isolated endovascular treatment. Therefore, hemiarch replacement was considered an appropriate strategy. This case highlights a potential limitation of the current surgical paradigm in which favorable remodeling is considered a surrogate for long-term safety. Even after apparent remodeling, patients with marked aortic tortuosity and unfavorable anatomic relationships between the descending aorta and esophagus may remain at risk for catastrophic late complications.

## Conclusions

This case demonstrates that fatal AEF may occur in a remodeled descending aorta after repair of acute type A IMH, independent of prosthetic materials or aneurysmal dilatation. Severe aortic tortuosity and persistence of the primary entry may represent important risk factors. Surgeons should remain vigilant for late esophageal complications, even in patients with favorable postoperative remodeling.

## Conflict of Interest Statement

The authors reported no conflicts of interest.

The *Journal* policy requires editors and reviewers to disclose conflicts of interest and to decline handling or reviewing manuscripts for which they may have a conflict of interest. The editors and reviewers of this article have no conflicts of interest.
